# Early post-weaning probiotic supplementation alters gut microbial community structure and predicted metabolic function in female juvenile mice

**DOI:** 10.3389/fendo.2026.1970147

**Published:** 2026-10-16

**Authors:** Xin Yuan, Huakun Shangguan, Ying Zhang, Ruimin Chen

**Affiliations:** Department of Pediatric Endocrinology, Genetics and Metabolism, Fuzhou First General Hospital Affiliated with Fujian Medical University, Fuzhou Children’s Hospital of Fujian, Fuzhou, China

**Keywords:** 16S rRNA, gut microbiota, PICRUSt2, probiotic, puberty, short-chain fatty acids

## Abstract

**Objective:**

To characterize the effects of early post-weaning multi-strain probiotic supplementation on gut microbiota composition, predicted metabolic function, and short-chain fatty acid (SCFA) profiles in female juvenile mice at the pubertal window.

**Methods:**

Female C57BL/6 mice were randomly assigned to probiotic (Pro) or control (Con) groups; after gavage-related losses, final sample sizes were Pro: n = 9 and Con: n = 6. The probiotic suspension contained Bifidobacterium longum, Lactobacillus bulgaricus, and Streptococcus thermophilus and was administered from postnatal day 21 to 27. Fecal samples were collected during the three days preceding vaginal opening for 16S rRNA gene sequencing (V3–V4 region, DADA2/ASV pipeline, SILVA 138.1 database), PICRUSt2 functional prediction, and targeted SCFA quantification. PERMANOVA with multivariate dispersion testing was used for beta-diversity analysis, and Benjamini-Hochberg correction was applied for multiple comparisons.

**Results:**

A total of 2,946 ASVs were identified from 15 fecal samples. Probiotic supplementation significantly decreased alpha diversity (Observed species, Chao1, ACE, and Shannon; all P < 0.05) and altered overall community structure (PERMANOVA: Bray-Curtis R² = 0.193, P = 0.013; Jaccard R² = 0.158, P = 0.008), with no difference in multivariate dispersion. No genus remained significant after FDR correction. At a nominally significant level, Prevotellaceae UCG-001 and an unclassified Bacteroidales genus were depleted, while Dubosiella, Parabacteroides, and an unclassified Lachnospiraceae genus were enriched. Akkermansia showed a trend toward enrichment (9.39% vs. 45.85%; ANCOM-BC2: log2FC = +2.15, P = 0.179, FDR = 0.395). PICRUSt2 identified 63 MetaCyc pathways at nominal significance, none surviving FDR correction (minimum FDR = 0.106). SCFA analysis revealed nominally significant reductions in caproic acid (P = 0.039) and elevations in acetic acid (P = 0.039), neither surviving FDR correction (FDR = 0.121). Two correlations remained significant at the exploratory FDR < 0.1 threshold: Prevotellaceae UCG-001–acetic acid (rho = −0.693) and Akkermansia–valeric acid (rho = −0.670).

**Conclusion:**

A brief post-weaning probiotic intervention significantly altered overall gut microbial community structure in female juvenile mice, with exploratory shifts in predicted functional capacity and a trend toward Akkermansia enrichment, without disrupting normal pubertal development. These findings should be considered exploratory given the small sample size.

## 1 Introduction

The gut microbiota constitutes a complex ecosystem that profoundly influences host physiology, including nutrient metabolism, immune function, and neuroendocrine signaling (1). Early life represents a critical developmental window during which the gut microbiota is highly plastic and susceptible to environmental modulation (2). Perturbations in early-life microbial colonization have been associated with long-term health consequences, including metabolic disorders, immune dysfunction, and altered neurodevelopment (3, 4).

Probiotics, defined as live microorganisms that confer health benefits when administered in adequate amounts (5), are widely used to modulate gut microbial composition. Among the most commonly employed probiotic species are Bifidobacterium longum, Lactobacillus bulgaricus, and Streptococcus thermophilus, which have demonstrated beneficial effects on gastrointestinal health, immune regulation, and metabolic homeostasis (6, 7). However, the extent to which short-term probiotic supplementation during the post-weaning period can reshape the gut microbiota and its metabolic output remains incompletely characterized.

The gut microbiota has been proposed to function as an “endocrine organ” capable of producing and metabolizing hormones and hormone-like substances (8, 9). Emerging evidence suggests potential crosstalk between the gut microbiome and the hypothalamic-pituitary-gonadal (HPG) axis, which regulates pubertal onset (10, 11). We previously reported that soy isoflavones promote earlier puberty onset in female mice, and that co-administration of probiotics obviates this effect while significantly altering fecal bacterial community composition (12). These findings raise the question of whether probiotic-induced microbial remodeling alone, in the absence of exogenous endocrine disruptors, is sufficient to influence pubertal timing.

In the present study, we performed a secondary analysis of the Pro (n = 9) and Con-observed (n = 6) groups from our previously described mouse cohort (12), focusing specifically on the effect of probiotic supplementation alone on the gut microbiota and its predicted functional capacity at the pubertal window. We administered a multi-strain probiotic formulation to female mice during the early post-weaning period (postnatal days 21–27) and characterized the gut microbiota composition, predicted functional capacity, and SCFA profiles at the pubertal window using a contemporary ASV-based DADA2 pipeline. While the original study characterized the effects of daidzein and probiotic co-administration on pubertal timing and gut microbiota composition, the specific comparison between probiotic-only and control animals was not reported. By establishing this baseline profile, we aim to provide foundational data for understanding gut microbiota–puberty interactions and to clarify the specific microbial and metabolic changes elicited by early-life probiotic intervention.

## 2 Materials and methods

### 2.1 Animals and experimental design

Specific pathogen-free inbred C57BL/6 pregnant mice (n = 20) were obtained from Minhou County Wu’s Experimental Animal Trade Co., Ltd (China). Mice were housed in pathogen-free plastic cages at Fujian Medical University under a 12-hour light/dark cycle (lights on at 07:00) with food and water available ad libitum. Female juvenile mice were weaned at postnatal day 21 (P21) and randomly assigned to probiotic (Pro, n = 10) or control (Con, n = 10) groups. Four control mice died during gavage operations in the first week of the intervention (P21–P27); no microbiological data were available for these animals, and no other exclusions were applied. The final sample sizes used for microbiome and metabolic analyses were Pro: n = 9 and Con: n = 6. Mice were group-housed at five animals per cage, with each cage containing animals from the same treatment group. The Pro group was housed in two cages and the Con group in two cages. A maximum of three mice from the same litter were allocated to each group. All procedures were approved by the Ethics Committee of Fuzhou Children’s Hospital of Fujian Medical University (approval number: 2019-26). The animals used in this study were part of a previously described cohort (12). The present analysis focused exclusively on the Pro and Con-observed groups, which received probiotic or vehicle, respectively, without daidzein exposure.

### 2.2 Probiotic preparation and administration

The probiotic treatment utilized a commercially available tablet formulation (Golden Bifid, Inner Mongolia Shuangqi Pharmaceutical Co., Ltd., China), each tablet containing live Bifidobacterium longum (≥0.5×10^7^ CFU), Lactobacillus bulgaricus (≥0.5×10^6^ CFU), and Streptococcus thermophilus (≥0.5×10^6^ CFU) per 0.5 g tablet. Tablets were ground into powder and dissolved in distilled water at 0.2 g/mL. Mice in the Pro group received 100 mg/kg/day via oral gavage from P21 to P27, corresponding to an estimated daily dose of approximately 1.0×10^6^ CFU/kg B. longum, 1.0×10^5^ CFU/kg L. bulgaricus, and 1.0×10^5^ CFU/kg S. thermophilus. Con mice received an equal volume of distilled water. Probiotic viability after preparation was not independently verified in this study.

### 2.3 Assessment of pubertal onset and tissue collection

Puberty onset was determined by daily observation of vaginal opening (VO) from P21 until occurrence, as previously described (12). Mice were sacrificed on the day of VO via intraperitoneal injection of sodium pentobarbital (150 mg/kg). Bilateral uteri and ovaries were dissected and weighed. Fecal samples were collected every morning at 09:00 during the three days preceding VO, pooled, and stored at −80 °C.

### 2.4 DNA extraction, 16S rRNA sequencing, and bioinformatics

Microbial community DNA was extracted using the MagPure Stool DNA KF kit B (Magen, China). The V3–V4 region of the bacterial 16S rRNA gene was amplified using primers 341F and 806R and sequenced on the Illumina MiSeq platform (BGI, Shenzhen, China) generating 2×300 bp paired-end reads.

Raw reads were processed using the DADA2 pipeline (v1.40.0) (13). Briefly, reads were quality-filtered (truncLen = c(260, 220), maxEE = c(2, 2), maxN = 0), denoised, and merged. Amplicon sequence variants (ASVs) were generated, and chimeras were removed using the consensus method. Taxonomic assignment was performed against the SILVA 138.1 reference database (14). Alpha diversity indices (Observed species, Chao1, ACE, Shannon, and Simpson) were calculated using the phyloseq package (15). Beta diversity was assessed using Bray-Curtis and Jaccard distances, with PERMANOVA (adonis2, 9,999 permutations) and multivariate dispersion testing (betadisper) performed using the vegan package (16). Differential abundance analysis was performed using ANCOM-BC2 (17), with Benjamini-Hochberg correction applied for multiple comparisons.

No rarefaction was applied prior to the main diversity analysis, which is consistent with current recommendations that rarefaction may reduce statistical power (18). Sequencing depth was comparable across samples (range: 18,290–35,646 reads per sample). A sensitivity analysis using rarefied data (rarefied to 18,290 reads per sample) yielded consistent results for both alpha diversity (Observed: P = 0.011; Chao1: P = 0.011; Shannon: P = 0.039) and beta diversity (PERMANOVA: R² = 0.190, P = 0.015). PICRUSt2 was used to predict MetaCyc pathway abundances from 16S rRNA data (19).

### 2.5 Short-chain fatty acid quantification

Fecal SCFAs (acetic, propionic, isobutyric, butyric, isovaleric, valeric, and caproic acids) were quantified using gas chromatography-mass spectrometry (Thermo Trace1300-Thermo TSQ9000, USA) in Selected Ion Monitoring mode. Data were processed using Tracefinder software (Thermo Fisher Scientific).

### 2.6 Statistical analysis

Statistical analysis were performed using R (v4.6.0). Group comparisons were performed using Wilcoxon rank-sum tests. Spearman’s rank correlation was used to assess associations between genus-level abundances and SCFA concentrations. Multiple alpha-diversity indices were examined; these should be considered supportive rather than independent inferential tests. Benjamini-Hochberg correction was applied for multiple comparisons where indicated. P < 0.05 was considered nominally significant; FDR < 0.05 (or FDR < 0.1 for correlation analysis) was considered significant after correction.

## 3 Results

### 3.1 Effect of probiotic supplementation on general development

At weaning (P21), body weights were comparable between the Pro and Con groups (7.51 ± 0.61 g vs. 7.39 ± 0.67 g, P > 0.05). No significant difference in body weight was observed at the time of vaginal opening (VO) (9.19 ± 0.83 g vs. 9.04 ± 0.86 g, P > 0.05). The average age at VO did not differ between groups (27.66 ± 1.09 d vs. 27.31 ± 0.97 d, P > 0.05). Uterine weights (8.43 ± 0.45 vs. 8.71 ± 1.07 mg, P > 0.05) and ovarian weights (11.70 ± 1.66 vs. 10.50 ± 1.44 mg, P > 0.05) were also comparable. These developmental parameters were consistent with our previously reported findings in the same mouse cohort (12).

### 3.2 Probiotic supplementation alters gut microbiota composition

A total of 366,554 high-quality sequences were obtained from 15 fecal samples (Pro: n = 9; Con: n = 6), with an average of 24,437 sequences per sample (range: 18,290–35,646). After denoising with DADA2 and removal of chimeras, 2,946 amplicon sequence variants (ASVs) were identified. Taxonomic assignment against the SILVA 138.1 database classified 98.8% of ASVs to phylum level, 92.0% to family level, and 48.4% to genus level.

At the phylum level, the dominant taxa were Bacteroidota, Verrucomicrobiota, and Firmicutes. Verrucomicrobiota was higher in the Pro group (32.26% vs. 5.44%), driven almost entirely by Akkermansia, whereas Bacteroidota was lower in the Pro group (46.75% vs. 69.48%) (**Figure 1**).

**Figure 1:**
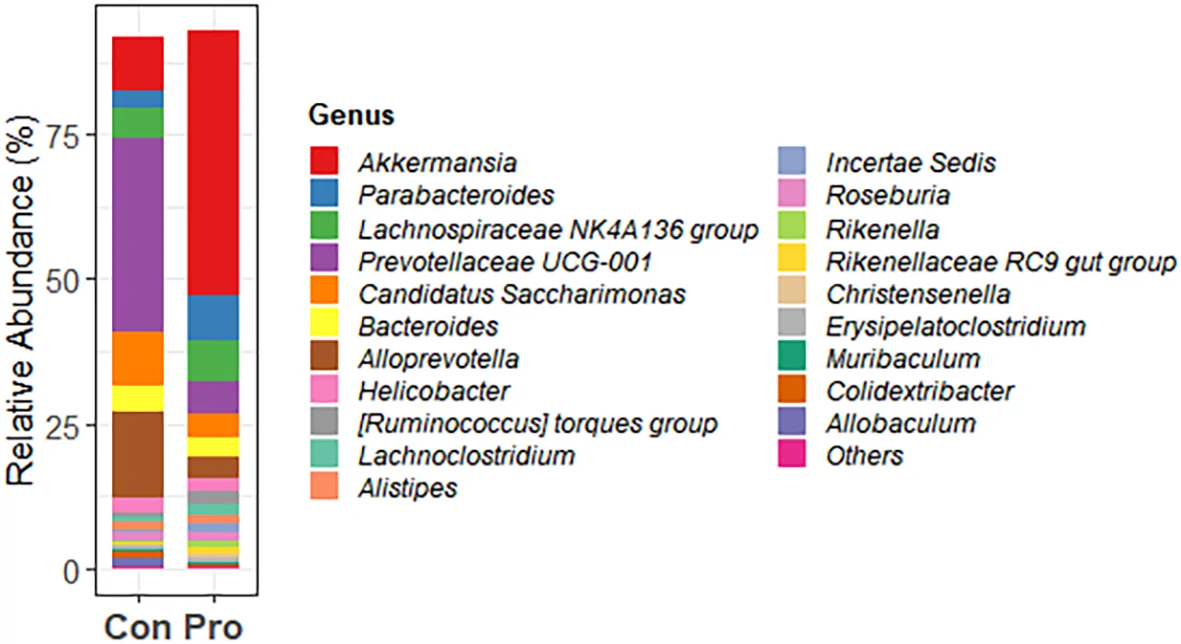
Gut microbiota composition at the genus level. Stacked bar plot showing mean relative abundance of the top 20 most abundant genera in the Con and Pro groups.

Alpha-diversity analysis revealed that the Pro group exhibited significantly lower Observed species (357 ± 45 vs. 428 ± 29, Wilcoxon test, P = 0.011), Chao1 (360 ± 45 vs. 438 ± 27, P = 0.008), and ACE indices (P = 0.011) compared with the Con group (**Figures 2A–C**). The Shannon index was also significantly reduced in the Pro group (P = 0.039; **Figure 2D**). The Simpson index did not differ significantly between groups (P = 0.068; **Figure 2E**).

**Figure 2:**
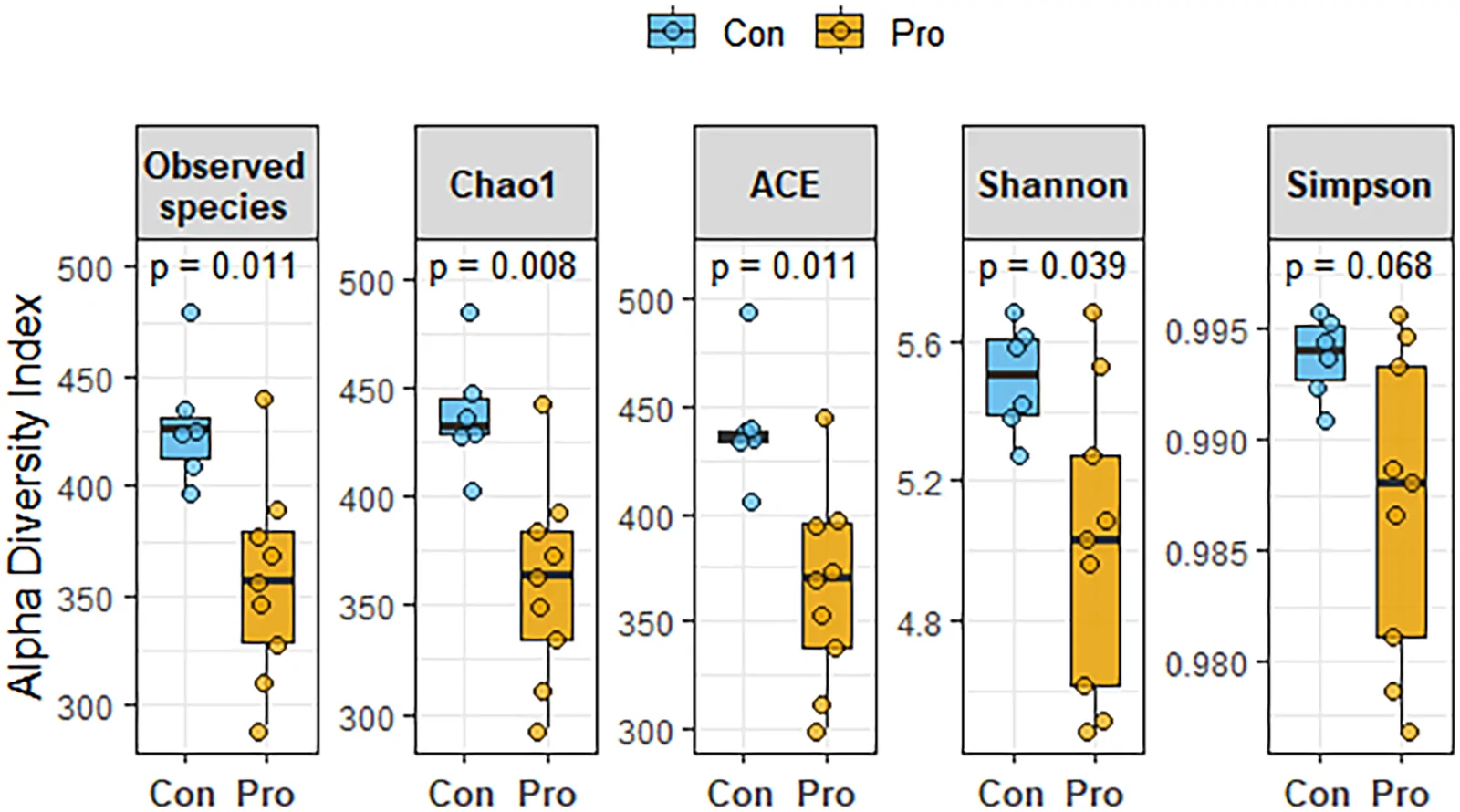
Alpha diversity indices in the Con and Pro groups.P values were determined by Wilcoxon rank-sum test.

Beta-diversity analysis, assessed by PERMANOVA based on Bray-Curtis and Jaccard distances, revealed significant differences in overall community structure between the Pro and Con groups (Bray-Curtis: R² = 0.193, P = 0.013; Jaccard: R² = 0.158, P = 0.008; **Figure 3**), although this result should be interpreted with caution given the confounding between treatment and cage. Multivariate dispersion analysis (betadisper) showed no significant difference in within-group variability, supporting (but not definitively confirming) the interpretation that the PERMANOVA result reflects compositional shifts rather than differences in dispersion (Bray-Curtis: P = 0.874; Jaccard: P = 0.904).

**Figure 3:**
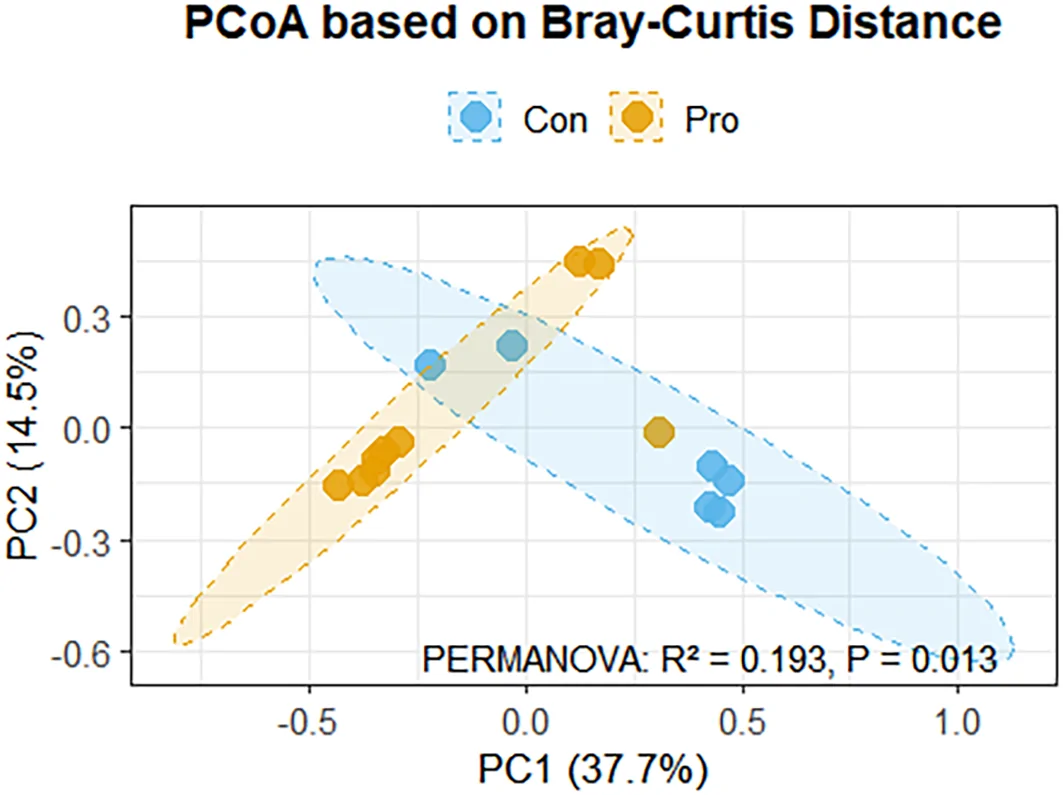
Principal coordinates analysis based on Bray-Curtis distance. PERMANOVA revealed significant differences in community structure between groups (R² = 0.193, P = 0.013). Ellipses represent 95% confidence intervals.

### 3.3 Differentially abundant taxa at the genus level

Differential abundance analysis using ANCOM-BC2 identified no genus that remained significant after Benjamini-Hochberg correction (FDR < 0.05). At a nominally significant level (raw P < 0.05), five genera showed differential abundance: Prevotellaceae UCG-001 (log2FC = −3.60, P = 0.006), an unclassified genus within the order Bacteroidales (log2FC = −0.44, P = 0.007), an unclassified genus within the family Lachnospiraceae (Incertae Sedis; log2FC = +2.25, P = 0.010), Dubosiella (log2FC = +2.42, P = 0.013), and Parabacteroides (log2FC = +1.98, P = 0.045) (**Supplementary Table S1**).

Among the eight genera examined by Wilcoxon rank-sum tests (**Figures 4A–H**), Akkermansia showed a trend toward enrichment in the Pro group (9.39 ± 14.93% vs. 45.85 ± 34.81%; ANCOM-BC2: log2FC = +2.15, P = 0.179, FDR = 0.395; Wilcoxon test: P = 0.016; **Figure 4A**). Paraprevotella was depleted in the Pro group (0.45 ± 0.23% vs. 0.057 ± 0.080%; ANCOM-BC2: log2FC = −1.26, P = 0.059, FDR = 0.314; Wilcoxon test: P = 0.003; **Figure 4G**). Prevotellaceae UCG-001 (ANCOM-BC2: P = 0.006; Wilcoxon: P = 0.016; **Figure 4B**) and Alloprevotella (ANCOM-BC2: P = 0.091; Wilcoxon: P = 0.052; **Figure 4F**) also showed trends toward depletion, while Lachnoclostridium (ANCOM-BC2: P = 0.077; Wilcoxon: P = 0.051; **Figure 4E**) and Parabacteroides (ANCOM-BC2: P = 0.045; Wilcoxon: P = 0.052; **Figure 4C**) showed trends toward enrichment in the Pro group. Dubosiella showed a nominal enrichment by ANCOM-BC2 (P = 0.013) but not by Wilcoxon test (P = 0.701; **Figure 4D**), and Bacteroides did not differ significantly between groups (Wilcoxon P = 0.216; **Figure 4H**). None of these comparisons survived FDR correction (**Supplementary Table S1**).

**Figure 4:**
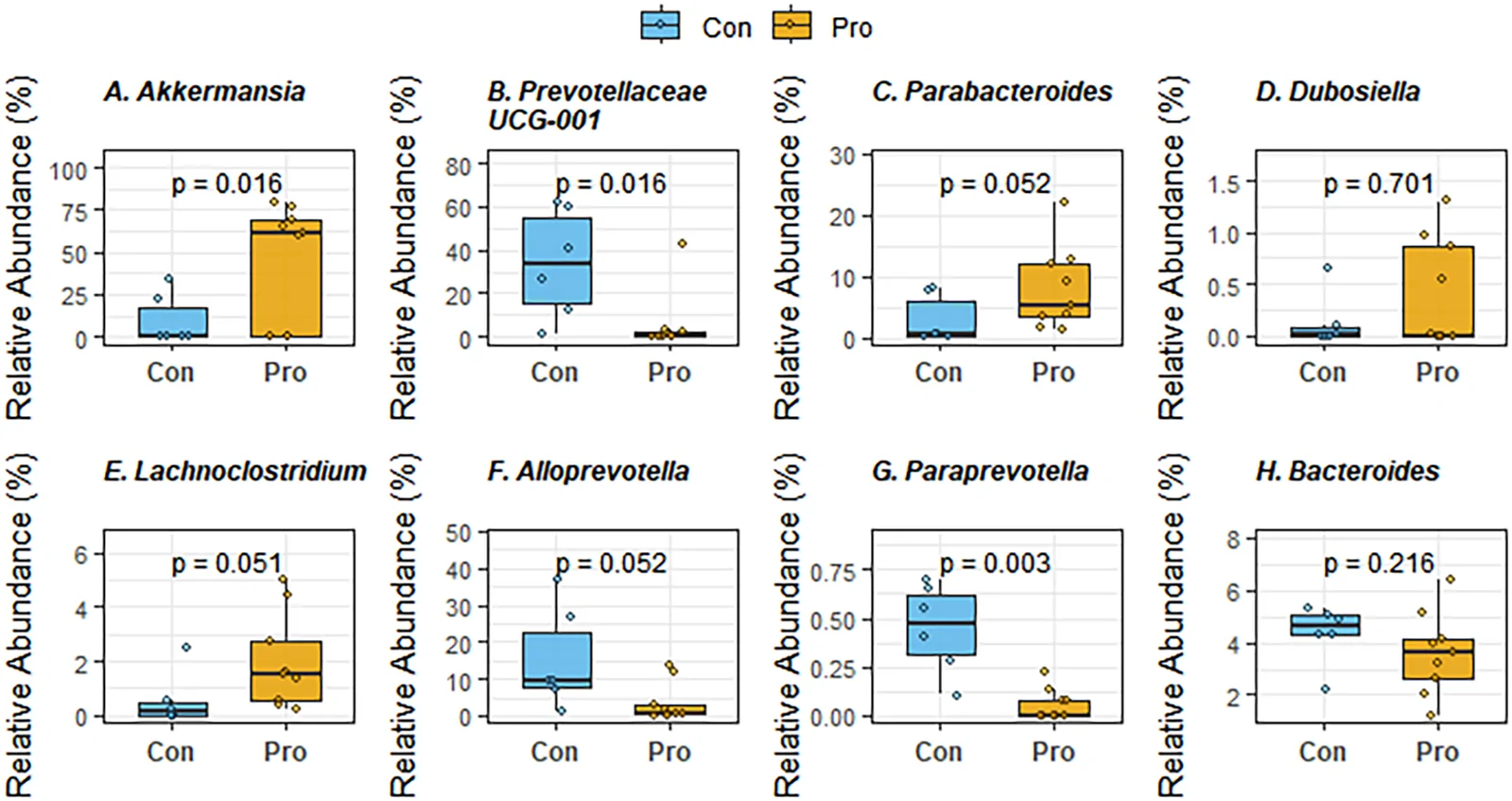
Relative abundance of key differentially abundant genera in the Con and Pro groups. Box plots showing **(A)** Akkermansia, **(B)** Prevotellaceae UCG-001, **(C)** Parabacteroides, **(D)** Dubosiella, **(E)** Lachnoclostridium, **(F)** Alloprevotella, **(G)** Paraprevotella, and **(H)** Bacteroides. P values shown in the panels were determined by Wilcoxon rank-sum test (raw P values; none survived FDR correction). Differential abundance was also assessed by ANCOM-BC2 (see Results and **Supplementary Table S1**).

### 3.4 Probiotic supplementation alters predicted functional potential

PICRUSt2 analysis was used to predict MetaCyc pathway abundances from the ASV data. At a nominally significant level (raw P < 0.05), 63 pathways differed between the Pro and Con groups; however, none survived Benjamini-Hochberg correction (minimum FDR = 0.106; **Supplementary Figure S1**; **Supplementary Table S2**). The complete list of differentially predicted pathways is provided in **Supplementary Table S2**.

### 3.5 Probiotic supplementation alters fecal SCFA profiles

Targeted metabolomics was employed to quantify seven SCFAs in fecal samples collected during the three days preceding vaginal opening. At a nominally significant level (raw P < 0.05), the Pro group exhibited reduced caproic acid concentration compared with the Con group (0.0081 ± 0.0019 vs. 0.0112 ± 0.0031 µg/mg, Wilcoxon test, P = 0.039; **Figure 5G**) and elevated acetic acid levels (0.105 ± 0.137 vs. 0.041 ± 0.037 µg/mg, P = 0.039; **Figure 5A**). Valeric acid showed a trend toward reduction in the Pro group (0.105 ± 0.151 vs. 0.207 ± 0.194 µg/mg, P = 0.052; **Figure 5F**). After Benjamini-Hochberg correction, none of these differences remained statistically significant (all FDR = 0.121; **Supplementary Table S3**). These differences also did not reach significance at the more permissive FDR < 0.1 threshold. No significant differences were observed for propionic acid (P = 0.680), butyric acid (P = 0.680), isobutyric acid (P = 0.175), or isovaleric acid (P = 0.953).

**Figure 5:**
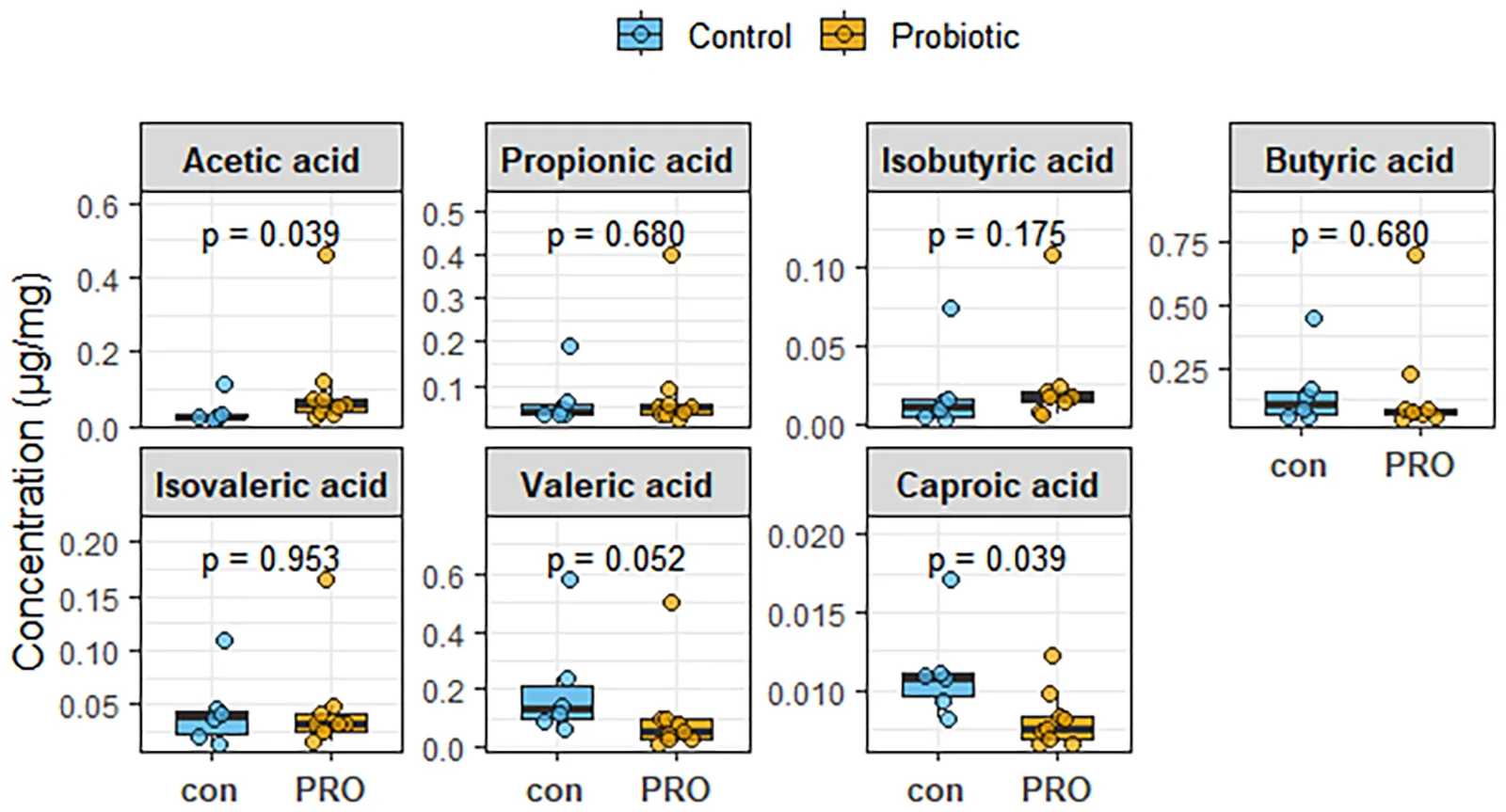
Fecal short-chain fatty acid (SCFA) concentrations in the Con and Pro groups.P values were determined by Wilcoxon rank-sum test (raw P values shown).

### 3.6 Correlation between key gut microbiota and SCFAs

Spearman’s rank correlation analysis was performed to explore the relationships between the key differentially abundant genera and SCFA concentrations (**Figure 6**; **Supplementary Table S4**). After Benjamini-Hochberg correction for 42 comparisons, two correlations remained significant at the exploratory FDR < 0.1 threshold. Prevotellaceae UCG-001 showed a negative correlation with acetic acid (rho = −0.693, P = 0.004, FDR = 0.066), and Akkermansia exhibited a negative correlation with valeric acid (rho = −0.670, P = 0.006, FDR = 0.066). At a nominally significant level (raw P < 0.05), additional associations included negative correlations between Oscillospira and acetic acid (rho = −0.657, P = 0.008), Klebsiella and valeric acid (rho = −0.610, P = 0.016), and Sutterella and acetic acid (rho = −0.561, P = 0.030), as well as positive correlations between Oscillospira and valeric acid (rho = 0.557, P = 0.031) and Prevotella and valeric acid (rho = 0.529, P = 0.043).

**Figure 6:**
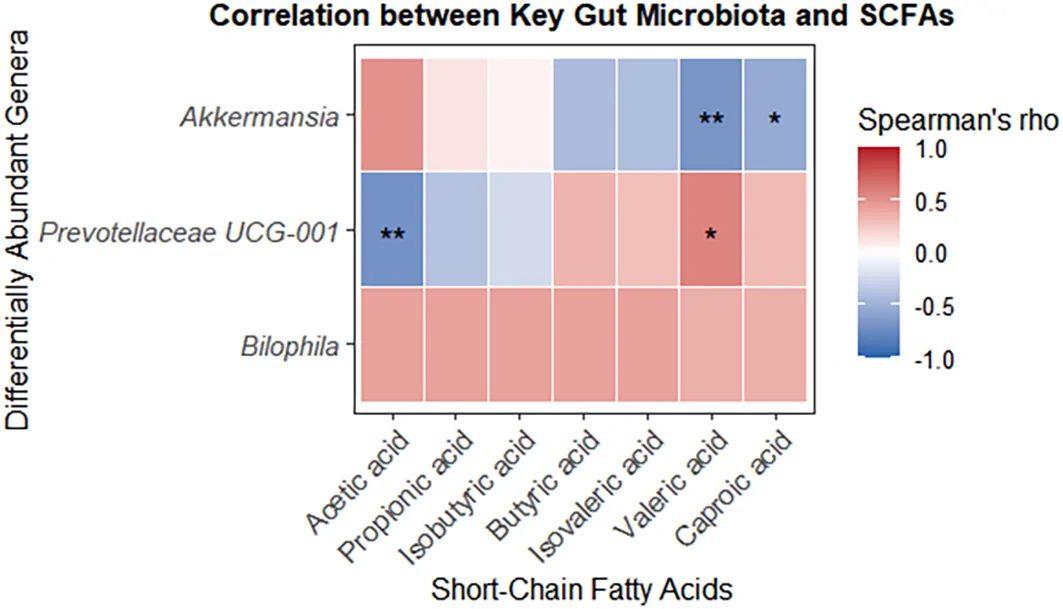
Correlation between key differentially abundant genera and SCFA concentrations. Heatmap showing Spearman correlation coefficients. Asterisks indicate nominally significant correlations (raw P < 0.05). Two correlations remained significant at the exploratory FDR < 0.1 threshold: Prevotellaceae UCG-001–acetic acid and Akkermansia–valeric acid. Asterisks indicate nominally significant correlations: *P < 0.05, **P < 0.01 (raw P values, uncorrected for multiple comparisons).

### 4 Discussion

In this study, we characterized the effects of a brief post-weaning multi-strain probiotic intervention on the gut microbiota and its metabolic output in female juvenile mice at the pubertal window. Using an ASV-based DADA2 pipeline and the SILVA 138.1 database, we found that probiotic supplementation significantly altered overall community structure and reduced alpha diversity, with exploratory shifts in predicted functional potential, despite no measurable effect on pubertal timing or reproductive organ development.

The most notable taxonomic observation was the trend toward Akkermansia enrichment in the probiotic group. Akkermansia muciniphila is a mucin-degrading commensal widely recognized for its beneficial effects on intestinal barrier integrity and metabolic health (20, 21). Although the Wilcoxon rank-sum test yielded a nominally significant P value (P = 0.016) for Akkermansia, this signal did not persist after FDR correction (FDR = 0.395). The high inter-individual variability in Akkermansia abundance should be noted when interpreting this trend. Nevertheless, the magnitude of the change and the consistency with previous studies showing that Bifidobacterium longum supplementation can promote Akkermansia colonization (22) suggest that this trend is biologically plausible. Larger studies are needed to confirm this observation.

The significant PERMANOVA results indicate that probiotic supplementation altered the overall community structure, not merely individual taxa. The absence of a significant dispersion effect supports, but does not definitively confirm, the interpretation that this difference reflects genuine compositional shifts, particularly given the small sample size and treatment-cage confounding. These results highlight the improved sensitivity of ASV-based approaches for detecting community-level changes.

At the genus level, several taxa showed nominally significant differences. Prevotellaceae UCG-001 and an unclassified Bacteroidales genus were depleted in the probiotic group, while Dubosiella, Parabacteroides, and an unclassified Lachnospiraceae genus were enriched. However, none of these differences survived FDR correction, and they should therefore be considered exploratory and hypothesis-generating rather than definitive. It should also be noted that genus-level classifications should be interpreted with caution, as the biological significance of a given genus can be context-dependent and cannot be resolved at the strain level using 16S rRNA data.

The reduction in alpha diversity observed in the probiotic group is consistent with the concept that probiotic administration can transiently reduce community richness by promoting the dominance of specific taxa (23). The increase in the relative abundance of Akkermansia and the concomitant decrease in Prevotellaceae UCG-001 suggest a shift toward a community dominated by Akkermansia and related taxa. This pattern has been reported in other probiotic intervention studies and may reflect competitive exclusion or niche occupation (24).

The PICRUSt2 functional predictions provide insight into the potential metabolic consequences of microbial remodeling. At a nominally significant level, 63 MetaCyc pathways differed between groups; however, none of these predicted changes survived FDR correction, and they should therefore be considered exploratory and hypothesis-generating. It is important to emphasize that PICRUSt2 provides predicted, not measured, functional capacity, and these inferences require validation through shotgun metagenomics or metatranscriptomics (25).

The observed changes in SCFA profiles should be interpreted with caution. Although acetic acid elevation and caproic acid reduction were nominally significant, they did not survive correction for multiple comparisons (FDR = 0.121), and they also did not reach significance at the more permissive FDR < 0.1 threshold, likely reflecting the limited statistical power of our small sample size. Nevertheless, the direction of these changes is biologically plausible: acetic acid is a primary fermentation product of Bifidobacterium and Akkermansia species (26, 27), both of which showed trends toward enrichment in the probiotic group. The significant negative correlation between Prevotellaceae UCG-001 and acetic acid (FDR = 0.066) provides additional exploratory support, as this genus was markedly depleted in the probiotic group. However, it should be emphasized that FDR < 0.1 is a permissive exploratory threshold rather than a conventional significance level, and correlation does not establish causation; both variables may be responding to the probiotic intervention or to other shared factors. These findings require validation in independent cohorts.

Under the conditions studied, probiotic-induced gut microbial remodeling alone did not detectably alter pubertal timing. Combined with our previous findings in the same cohort, this suggests that the puberty-related effects of probiotics may depend on co-exposure to exogenous endocrine-active compounds, although this hypothesis requires further validation. Given the small sample size, the absence of circulating hormone measurements, and the single time point of assessment, we cannot exclude the possibility that a modest effect on pubertal development was missed. Future studies employing longer intervention periods, multiple sampling time points, and direct measurement of circulating gonadotropins and estradiol would be valuable to further clarify this relationship (28, 29).

Several limitations should be acknowledged. First, the small sample size (n = 6–9 per group) limited statistical power, and no genus-level differences survived FDR correction. Second, animals were group-housed with cage mates belonging to the same treatment group (two cages per group), which means that treatment and cage are substantially confounded. Individual mice cannot be considered fully independent experimental units, and the possibility of pseudoreplication cannot be excluded. With only two cages per treatment group, a reliable cage-level treatment effect cannot be robustly estimated. The PERMANOVA results should therefore be interpreted with caution, and no causal inference regarding the treatment effect on microbial community structure can be drawn. Third, the relatively low genus-level assignment rate (48.4%) reflects the inherent limitations of the V3–V4 region and current reference databases. Fourth, PICRUSt2 provides predicted rather than measured functional capacity, and probiotic colonization was not directly assessed. Finally, no circulating hormones were measured, so a modest effect on pubertal development cannot be excluded. No *a priori* power calculation was performed, and qPCR validation of the key taxa was not conducted due to limited sample availability.

## 5 Conclusion

In summary, using a contemporary ASV-based DADA2 pipeline, we demonstrate that a brief post-weaning multi-strain probiotic intervention significantly altered overall gut microbial community structure and was associated with exploratory differences in predicted functional capacity in female juvenile mice, with Akkermansia showing a trend toward enrichment and Prevotellaceae UCG-001 showing a trend toward depletion. These compositional changes were accompanied by exploratory shifts in SCFA metabolism. Despite these microbial effects, probiotic supplementation did not detectably influence pubertal timing or reproductive organ development under the conditions studied. Given the small sample size and the exploratory nature of the taxonomic and metabolic findings, these results should be interpreted with caution and warrant confirmation in larger, adequately powered studies. This work provides a baseline characterization of probiotic-induced microbial remodeling at the pubertal window using current analytical standards.

## Data Availability

The datasets presented in this study can be found in online repositories. The names of the repository/repositories and accession number(s) can be found in the article/**Supplementary Material**.
