# Supplementary material for: Early post-weaning probiotic supplementation alters gut microbial community structure and predicted metabolic function in female juvenile mice
Source: Front Endocrinol (Lausanne). 2026 Oct 16;17:1970147. doi: 10.3389/fendo.2026.1970147 (PMC13719360; doi:10.3389/fendo.2026.1970147)
Supplement: Supplementary file 1 [file Supplementaryfile1.docx]

**Figure S1.** Differentially predicted MetaCyc pathways identified by PICRUSt2. Bar plot showing the top 20 pathways ranked by raw P value (all raw P < 0.05). The x-axis represents log2 fold change (Pro/Con). Orange bars indicate pathways enriched in the Pro group; blue bars indicate pathways enriched in the Con group. None of these pathways survived Benjamini-Hochberg correction (minimum FDR = 0.106). These results should be considered exploratory.

**Table S1.** Differentially abundant genera identified by ANCOM-BC2 (nominally significant, raw P < 0.05; none survived FDR correction).

| **Genus** | **Mean_Con (%)** | **SD_Con** | **Mean_Pro (%)** | **SD_Pro** | **log2FC** | **P value** | **FDR** |
| --- | --- | --- | --- | --- | --- | --- | --- |
| Prevotellaceae UCG-001 | 33.72 | 24.84 | 5.56 | 14.05 | −3.60 | 0.006 | 0.141 |
| Unclassified Bacteroidales | 2.01 | 0.22 | 2.13 | 0.42 | −0.44 | 0.007 | 0.141 |
| Incertae Sedis (Lachnospiraceae) | 0.41 | 0.79 | 1.41 | 1.58 | +2.25 | 0.010 | 0.141 |
| Dubosiella | 0.13 | 0.26 | 0.41 | 0.52 | +2.42 | 0.013 | 0.145 |
| Parabacteroides | 2.91 | 3.86 | 7.90 | 6.81 | +1.98 | 0.045 | 0.314 |

**Note:** ANCOM-BC2 was used for differential abundance analysis with Benjamini-Hochberg correction. No genus remained significant at FDR < 0.05. Genera shown in Figure 4 but not listed here (Akkermansia, Lachnoclostridium, Alloprevotella, Bacteroides) did not reach nominal significance by ANCOM-BC2.

**Table S2.** PICRUSt2-predicted MetaCyc pathway abundances in the Con and Pro groups (top 20 by raw P value).

| **Pathway** | **Description** | **log2FC** | **P value** | **FDR** |
| --- | --- | --- | --- | --- |
| PWY-5180 | toluene degradation I (aerobic) (via o-cresol) | −3.47 | 0.0016 | 0.106 |
| PWY-5415 | catechol degradation I (meta-cleavage pathway) | −3.55 | 0.0016 | 0.106 |
| PWY-5419 | catechol degradation to 2-hydroxypentadienoate II | −2.66 | 0.0016 | 0.106 |
| PWY-5420 | catechol degradation II (meta-cleavage pathway) | −3.28 | 0.0016 | 0.106 |
| PWY-5430 | meta cleavage pathway of aromatic compounds | −3.25 | 0.0016 | 0.106 |
| PWY-7480 | 2,3-dihydroxybenzoate degradation | −3.13 | 0.0016 | 0.106 |
| PWY-7316 | dTDP-N-acetylviosamine biosynthesis | −1.37 | 0.0018 | 0.106 |
| PWY-5989 | stearate biosynthesis II (bacteria and plants) | −1.60 | 0.0039 | 0.202 |
| PWY-5005 | biotin biosynthesis II | +4.14 | 0.0052 | 0.212 |
| GLYOXYLATE-BYPASS | glyoxylate cycle | −1.36 | 0.0056 | 0.212 |
| PWY0-1479 | tRNA processing | −1.71 | 0.0056 | 0.212 |
| ARGSYN-PWY | L-arginine biosynthesis I (via L-ornithine) | +0.45 | 0.0157 | 0.263 |
| HISTSYN-PWY | L-histidine biosynthesis | +0.25 | 0.0216 | 0.263 |
| DENITRIFICATION-PWY | nitrate reduction I (denitrification) | −3.00 | 0.0109 | 0.263 |
| PPGPMET-PWY | ppGpp metabolism | −0.31 | 0.0113 | 0.263 |

**Note:** Wilcoxon rank-sum test with Benjamini-Hochberg correction. At a nominally significant level (raw P < 0.05), 63 pathways differed between groups; none survived FDR correction (minimum FDR = 0.106). The complete list of 415 pathways is provided in the Supplementary Data file.

**Table S3.** Fecal short-chain fatty acid (SCFA) concentrations in the Con and Pro groups.

| **SCFA** | **Mean_Con (µg/mg)** | **SD_Con** | **Mean_Pro (µg/mg)** | **SD_Pro** | **P value** | **FDR** |
| --- | --- | --- | --- | --- | --- | --- |
| Acetic acid | 0.041 | 0.037 | 0.105 | 0.137 | 0.039 | 0.121 |
| Propionic acid | 0.065 | 0.061 | 0.088 | 0.119 | 0.680 | 0.793 |
| Isobutyric acid | 0.020 | 0.027 | 0.026 | 0.032 | 0.175 | 0.307 |
| Butyric acid | 0.158 | 0.150 | 0.159 | 0.211 | 0.680 | 0.793 |
| Isovaleric acid | 0.044 | 0.035 | 0.046 | 0.046 | 0.953 | 0.953 |
| Valeric acid | 0.207 | 0.194 | 0.105 | 0.151 | 0.052 | 0.121 |
| Caproic acid | 0.011 | 0.003 | 0.008 | 0.002 | 0.039 | 0.121 |

**Note:** Wilcoxon rank-sum test with Benjamini-Hochberg correction. No SCFA remained significant after FDR correction. Raw P values are shown for descriptive purposes.

**Table S4.** Spearman correlations between key genera and SCFA concentrations (FDR < 0.1).

| **Genus** | **SCFA** | **rho** | **P value** | **FDR** |
| --- | --- | --- | --- | --- |
| Prevotellaceae UCG-001 | Acetic acid | −0.693 | 0.004 | 0.066 |
| Akkermansia | Valeric acid | −0.670 | 0.006 | 0.066 |

**Note:** Spearman's rank correlation with Benjamini-Hochberg correction for 21 comparisons. Only correlations with FDR < 0.1 are shown. The complete list is provided in the Supplementary Data file.
